# Clinical tool for disease phenotyping in granulomatous lung disease

**DOI:** 10.1371/journal.pone.0188119

**Published:** 2017-11-16

**Authors:** Lori J. Silveira, Matthew Strand, Michael V. Van Dyke, Margaret M. Mroz, Anna V. Faino, Dana M. Dabelea, Lisa A. Maier, Tasha E. Fingerlin

**Affiliations:** 1 Division of Biostatistics and Informatics, National Jewish Health, Denver, Colorado, United States of America; 2 Colorado School of Public Health, University of Colorado Anschutz Medical Campus, Aurora, Colorado, United States of America; 3 Colorado Department of Public Health and Environment, Denver, Colorado, United States of America; 4 Division of Environmental and Occupational Health, National Jewish Health, Denver, Colorado, United States of America; 5 Department of Medicine, University of Colorado Anschutz Medical Campus, Aurora, Colorado, United States of America; 6 Center for Genes, Environment and Health, National Jewish Health, Denver, Colorado, United States of America; University of Alabama at Birmingham, UNITED STATES

## Abstract

**Background:**

Exposure to beryllium may lead to granuloma formation and fibrosis in those who develop chronic beryllium disease (CBD). Although disease presentation varies from mild to severe, little is known about CBD phenotypes. This study characterized CBD disease phenotypes using longitudinal measures of lung function.

**Methods:**

Using a case-only study of 207 CBD subjects, subject-specific trajectories over time were estimated from longitudinal pulmonary function and exercise-tolerance tests. To estimate linear combinations of the 30-year values that define underlying patterns of lung function, we conducted factor analysis. Cluster analysis was then performed on all the predicted lung function values at 30 years. These estimates were used to identify underlying features and subgroups of CBD.

**Results:**

Two factors, or composite measures, explained nearly 70% of the co-variation among the tests; one factor represented pulmonary function in addition to oxygen consumption and workload during exercise, while the second factor represented exercise tests related to gas exchange. Factors were associated with granulomas on biopsy, exposure, steroid use and lung inflammation. Three clusters of patients (n = 53, n = 59 and, n = 95) were identified based on the collection of test values. Lower levels of each of the factor composite scores and cluster membership were associated with baseline characteristics of patients.

**Conclusions:**

Using factor analysis and cluster analysis, we identified disease phenotypes that were associated with baseline patient characteristics, suggesting that CBD is a heterogeneous disease with varying severity. These clinical tools may be used in future basic and clinical studies to help define the mechanisms and risk factors for disease severity.

## Introduction

Chronic beryllium disease (CBD) is caused by inhalation of beryllium particulate that initiates an immune response in the lung. While many studies have examined beryllium’s ability to initiate an immune response and cause granuloma formation [1–12], there are many questions remaining regarding CBD clinical manifestations. Currently, there is a lack of understanding of the determinants of disease progression and a corresponding lack of consensus regarding useful definitions of severity. Although there have been several genetic polymorphisms and genes whose expression have been associated with phenotypes consistent with more severe disease [9–14], there have been no studies to date that have attempted to comprehensively characterize or define the phenotypes of CBD. Such characterization has the potential to allow development of individualized strategies of follow-up and care based on disease course and may enable more thorough examination of risk factors for the different phenotypes of this disease.

In existing studies of CBD disease progression, pulmonary function tests (PFT) and exercise testing results were used as surrogate indicators of disease severity.[13, 15–17] Because CBD patients are usually seen at least biennially after diagnosis, physicians can follow disease progression over time to make decisions regarding treatment. The PFT and exercise testing obtained are multidimensional: measurements are taken over time, with each one of these tests consisting of multiple measures. Our current understanding of CBD suggests that patients may demonstrate different phenotypes over time, with some developing severe airflow limitation and others severe reduction in gas exchange,[17, 18] but no existing studies have used the full complement of multiple longitudinal measures to examine these phenotypes. Objective criteria for characterizing disease phenotypes would allow for consistency in disease assessment and characterization for clinicians and for researchers. The purpose of this study was to characterize longitudinal disease phenotypes in CBD using data reduction techniques to simultaneously consider all the multi-dimensional data available over time and across measures. Our working hypothesis was that by using longitudinal pulmonary and exercise test data, we could define different phenotypes in CBD and begin to have definitions of disease severity that could be evaluated over time.

## Methods

### Overview of approach

Longitudinal lung function data were used to obtain estimates of the progression of disease for each study subject. These estimates were used in factor and cluster analysis, in an attempt to identify new phenotypic measures (factors) or classifications (cluster) of CBD disease. We examined the consistency among the results of each approach by evaluating associations between the few known predictors of severity available and the newly-developed factors and clusters.

### Overview of study population, design, and case definition

This was a case-only study. Subjects were patients seen at National Jewish Health (NJH), all of whom provided informed consent according to a protocol reviewed by the Human Subjects Institutional Review Board at NJH (HS2374). A case of CBD had evidence of beryllium sensitization based on two or more positive blood lymphocyte proliferation test (BeLPT) results as well as one of the following: 1) granulomas on a lung biopsy, 2) a positive bronchoalveolar lavage (BAL) lymphocyte proliferation test and BAL lymphocytes percentages greater than 15%, or 3) chest radiography indicating an abnormal profusion score consistent with CBD. For analysis purposes, diagnosis type was dichotomized, with groups 2) and 3) combined due to small numbers of each type. We included all CBD cases seen at NJH who consented for research and who had at least 3 visits to allow estimation of linear slopes (trajectories) over time.

### SSP-PCR determination of the HLA-DPB1 alleles

Genomic DNA was prepared from peripheral blood cells. HLA-DPB1 genotyping was performed with blinding to the subject’s disease status using single specific primer polymerase chain reaction (SSP-PCR) methodology developed by Welsh and Bunce. Based on alleles, a subject was classified as Glu69 positive or negative.

### Outcome definitions

Clinical evaluations were completed on initial assessment as well as during follow-up over time at NJH. These evaluations included pulmonary function and exercise physiology testing, as well as chest radiography. We included the following measures: forced expiratory volume in one second (FEV1), forced vital capacity (FVC), diffusion capacity(DLCO), total lung capacity (TLC), partial pressure of oxygen at rest (PaO_2_r) and maximal exercise (PaO_2_m), Arterial-alveolar gradient for oxygen at rest ((A-a)O_2_rest) and at maximal exercise((A-a)O_2_max), oxygen consumption at maximal exercise (VO_2_m), and maximum workload(WLM). Descriptions of procedures for these tests have been previously published.[17, 18] Per clinical practice, after having two positive BeLPTs, patients normally are evaluated on an annual or biennial basis as part of a surveillance program prior to diagnosis, and on an annual basis after a CBD diagnosis. All CBD subjects’ data available prior to and after diagnosis were included in this study.

### Patient data

Information from medical records, such as steroid use and general demographic information, was extracted along with all longitudinal results from clinical evaluations. Steroid use was coded as “Ever” if the subject had ever been prescribed steroids for CBD and “Never” if they had not. Subjects’ most recent chest x-ray images were used in this analysis and were coded “Abnormal” if the subject’s International Labor Organization (ILO) classification of chest radiograph’s profusion score was 0/1 or higher and “Normal” otherwise. For BAL lymphocyte percentage, we compared the lymphocytes from the subjects’ first visit and last visit in order to assess whether subjects’ BAL lymphocyte percentages at different time points were associated in any way with our disease severity measures.

### Statistical analysis

Population characteristics were summarized using means and standard deviations for continuous variables; counts and frequencies were used to summarize categorical variables. To capture longitudinal information on disease course, we estimated longitudinal trajectories of several clinically-relevant lung-function variables using linear mixed-effects models [19]. Patient-specific slopes (trajectories over time) and intercepts (starting values at first beryllium exposure) were estimated via random intercept and slope terms. The time variable was defined as the interval from first beryllium exposure to most recent visit date. Covariates for age at testing (time-varying), sex, and race were also included in the model. Time-specific predicted values for each subject were calculated for each lung-function variable using the fitted estimates from the model for that variable; the primary time point of interest was 30 years since first exposure since it captures both the patient starting point (intercept) and trajectory (slope) estimates. The longitudinal models were fit using SAS 9.2.[20]

To attempt to identify distinct groups of patients, or phenotypes, demonstrating a similar pattern of lung function at 30 years after exposure within each group, cluster analysis was performed on all the predicted lung function values at 30 years. We used the “kError” method, which accounts for the fact that these 30-year time points are not observed, but rather, predicted values.[21] To estimate quantitative summary measures of the lung function values, linear combinations of the 30-year values that define underlying patterns of lung function were calculated based on a factor analysis. In contrast to a cluster analysis, which assigns each individual to one cluster, factor analysis produces a quantitative estimate for each factor for each person. Factor analysis reduces a set of variables to a smaller set of factors in an attempt to better represent the data for more effective reasoning, relevant insights, or better visualization. Conceptually, each person is placed on a continuum for each of the factors rather than forcing assignment to a particular group. After the initial extraction of factors using the “factanal”function (https://www.rdocumentation.org/packages/FAiR/versions/0.4-15/topics/Factanal R version 3.1.1); which uses maximum likelihood estimation) in R, a varimax rotation was chosen to simplify interpretation by creating orthogonal (independent) factors.

Examination of the impact of using predicted values (i.e. estimated rather than known) on identification of factors was evaluated through simulation since there is no analogous method to directly account for the uncertainty as is available in the kError method for cluster analysis. We simulated 25 replicates of multivariate normal data using the means, standard errors and correlation structure observed in our data among the 30-year predicted values to determine the consistency of the factors identified across data sets of similar structure. We similarly simulated 25 replicates generated with *random* covariance matrices (i.e. ignoring the observed correlation structure among lung function variables) but retaining the observed means and standard errors. We conducted the factor analysis for each replicate.

We tested for association between cluster group membership or factor composite scores with demographic, clinical and exposure characteristics measured at baseline using chi-squared tests for cluster membership and ANOVA for factor composite scores.

Finally, to compare the results of the factor analysis and cluster analysis to associations we would observe by testing each lung function or exercise test separately, we constructed models to test whether steroid use was associated with the longitudinal trajectory of each lung function variable and whether steroid use was significantly associated with both Factor composite score and Cluster membership. We similarly tested for an association between each longitudinal trajectory and having a job as a beryllium machinist (a surrogate measure for higher exposure). These models were the same as described above, with the addition of the specific explanatory variable.

## Results

### Two factors associated with lung function measures

Table 1 displays the characteristics of the 207 participants; we observed an average of 8 follow-up visits for each patient (mean of 8.6, standard deviation 5.6). Two factors, or underlying combinations of variables that captured covariation among all the 30-year lung function and exercise test variables, were identified in the factor analysis [22, 23] of the standardized individual estimates of each lung function and exercise test measure at 30 years from first exposure; 39% of the covariation in the data was explained by Factor 1, while 29% of the covariation in the data was explained by Factor 2 (Table 2). The factor loads, or coefficients for each lung function or exercise test variable, provide information on the relative importance and directions of effect for each measure that contributed to the factor. The factor composite score for each individual was the linear combination of the 30-year estimates using the factor loads; a lower value for the factor reflected more severe disease. The composite score for Factor 1 predominately reflected PFT measures as the coefficients are largest for those measures, and is related to airflow and lung volumes and their correlation with exercise, including both VO_2_m and WLM. The composite score for Factor 2 consisted of exercise test measures that reflect gas exchange. Of note is the stronger relationship of VO_2_m and WLM with the PFT measures than with the other exercise variables. These results were highly consistent in terms of the number of Factors, the variables contributing to them and the average coefficients for each variable in the data simulated to have the same structure as our data. In contrast, in replicate data sets that ignored the observed correlation structure among lung function variables, there were no factor structures that were the same as those observed in our data. These results indicate that the Factor analysis findings are reproducible and driven by the correlation between the lung function variables.

**Table 1:** CBD patient population demographics and clinical results (N = 207).

**Characteristic**	**Number (%)**
Male n (%)	162 (78.3%)
Caucasian n (%)	166 (80.2%)
Smoking Status–Ever Smoker n (%)	100 (48.3%)
	**Mean (SD)**
Age at diagnosis	53.6 (10.6)
**Diagnosis**	**Number (%)**
By granulomas on biopsy n (%)	164 (79.2%)
By positive BAL LPT/>15% lymphocytes n (%)	38 (18.4%)
By radiography n (%)	5 (2.4%)
**PFT and Exercise Outcomes at Baseline**	**Mean (SD)**
FEV_**1**_% predicted	90.4 (17.7)
FVC % predicted	88.1 (15.4)
TLC % predicted	103.1 (14.8)
DLCO % predicted	92.7 (21.5)
PaO2r (mmHg)	70.6 (8.9)
PaO2m (mmHg)	75.4 (10.9)
(A-a)O2 rest (mm Hg)	11.1 (7.3)
(A-a)O2 max (mmHg)	17.9 (13.4)
WLM—Maximum work Load Achieved (watts)	164.9 (50.0)
VO_2_ m (Liters/minute)	1.9 (0.53)
**Exposure and Visit Characteristics**	**Mean (SD)**
Time from first exposure to diagnosis	22.8 (10.9)
Average exposure years*	15.2 (10.3)
Average number of visits	7.3 (5.6)

*If exposure end date unknown, estimated as date of diagnosis

**Table 2:** Factors and loadings for standardized outcome estimates at 30 years.

Measure	Factor 1 Coefficient * PFT, VO_2_max +WLM	Factor 2 Coefficient * Exercise Test
**FEV1**	0.872	-
**FVC**	0.980	-
**TLC**	0.772	-
**DLCO**	0.640	-
**PaO**_**2**_**r**	-*	0.687
**PaO**_**2**_ **m**	-	0.885
**(A-a)O**_**2**_**rest**	-	0.696
**(A-a)O**_**2**_**max**	-	0.892
**VO**_**2**_ **m**	0.621	
**WLM**	0.632	
**% co-variation explained by factor across all measures**	0.39	0.28

*Coefficients less than 0.50 were excluded to focus factor development; indicated by “-”on strongest distinguishing variables.

### Factors associated with granulomas on biopsy, exposure, steroid use and lung inflammation

We next examined associations between the Factors for each person and demographic, clinical and genetic features (Table 3) measured at baseline. Patients diagnosed with granulomas on biopsy had lower Factor 2 (exercise test) composite scores on average (p = 0.001), indicating poorer exercise gas exchange, compared to those diagnosed by lavage or radiography. Higher exposure to beryllium was also associated with lower Factor 2 (exercise test) composite scores on average (p = 0.003). Both Factor 1 (PFT, VO_2_m + WLM) and Factor 2 (exercise test) composite scores were lower on average (p = 0.0001 Factor 1, p<0.0001 Factor 2) for patients who had ever been treated with steroids compared to those who had never been treated. Similarly, patients who had abnormal chest x-rays had lower composite scores on average (p<0.0001, Factor 1, p<0.0001 Factor 2) compared to those with a normal chest x-ray. Finally, baseline and most recent BAL lymphocyte percentages were negatively associated with Factor 2 (exercise test) (p<0.0001); higher BAL lymphocyte percentage was associated with lower Factor 2 composite scores on average.

**Table 3:** Association between patient characteristics and factor composite scores.

	Factor 1 PFT, VO_2_m + WLM*	P-Value	Factor 2 Exercise Test *	P-Value
**Sex**		0.71		0.93
Male	0.04 (3.5)		0.01 (2.5)	
Female	-0.15 (2.9)		-0.04 (3.4)	
**Race**		0.56		0.96
Caucasian	0.02 (3.4)		-0.005 (2.6)	
Other	-0.09 (3.1)		0.02 (3.4)	
**Age of Diagnosis**	-0.04	0.58	-0.01	0.84
**Diagnosis Type**		0.36		0.001
By biopsy granulomas	-0.02 (3.5)		-0.11(2.8)	
By bronchoalveolar lavage or chest x- ray	0.48 (2.3)		1.3 (1.7)	
**Ever Smoked**				
Yes	-0.21 (3.3)	0.38	-0.02 (2.3)	0.91
No	0.20 (3.4)		0.02 (3.1)	
**Steroids Ever**				
Yes	-1.58 (3.5)	0.0002	-1.82 (3.3)	0.0005
No	0.48 (3.2)		0.60 (2.3)	
**Lymphocyte Percent**^§^				
Closest to Diagnosis	-0.10	0.16	-0.31	<0.0001
Most Recent	-0.10	0.20	-0.36	<0.0001
**Abnormal CXR**				
Yes	-3.0 (3.2)	<0.0001	-4.4 (3.5)	<0.0001
No	.35 (3.2)		0.56 (2.0)	
**Exposure**				
Years	0.12	0.12	-0.02	0.77
Machinists	-0.09 (2.6)	0.89	0.17 (2.7)	0.68
Non-Machinists	-0.16 (3.5)		-0.03 (2.8)	
High exposure	-0.27 (3.2)	0.21	-0.18 (2.9)	0.003
Low Exposure	0.58 (4.4)		1.1 (1.8)	
**High-Risk E69 Allele**		0.83		0.92
Have an E69 allele	-0.12 (2.3)		-0.05 (2.8)	
No E69 alleles	0.12 (4.5)		-0.12 (3.5)	

*Mean (SD) of factor composite score for categorical group or correlation between measure and composite score.

^§^Natural logarithm transformed

### Three clusters identified

**Fig 1:**
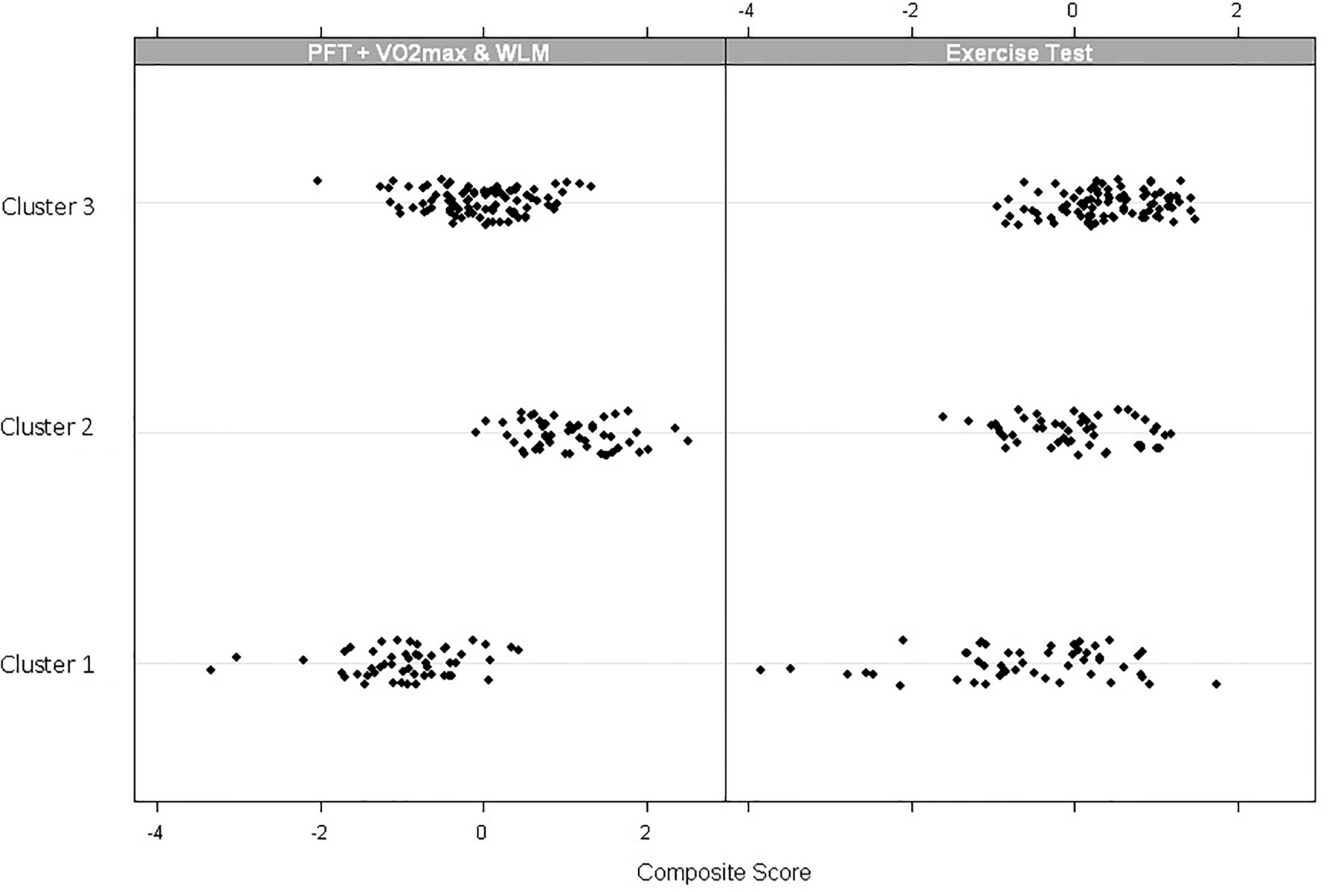
Comparison of factors between clusters. Cluster 1 had significantly lower composite scores than Clusters 2 or 3 (p<0.0001). Cluster 3 had significantly lower PFT, VO_2_m + WLM composites than Cluster 2 (p<0.0001).

Using the same individual estimates in the factor analysis for lung function and exercise test measure at 30 years from first exposure, three clusters were identified that minimized the within-cluster variance.[23] There were 53 subjects in Cluster 1, 59 subjects in Cluster 2, and 95 subjects in Cluster 3; these cluster assignments were consistent in 25 replicates of the analysis using random initial assignments of each person to a cluster. The characteristics of each cluster are shown in Table 4; as expected, the mean of each of the individual 30-year estimates differed across the three clusters (all p <0.0001). Tests of association between cluster membership and lung function variables demonstrate worse lung function for those in Cluster 1 compared to the other clusters. The average 30-year estimates were significantly lower for most PFT and exercise test parameters, except A-a gradient, which was higher (indicative of worse disease), in Cluster 1 compared to Cluster 3 (all p<0.0001). Two of the exercise test parameters (VO_2_m and WLM) were statistically different between Clusters 2 and 3 (both p<0.0001); these are the same two measures that were more correlated with the PFT measures than the exercise test measures in the factor analysis. Individuals in Cluster 1 had significantly lower Factor 1 (PFT + VO_2_max and WLM) and Factor 2 (exercise test) composite scores on average compared to Clusters 2 and 3 (p<0.0001, <0.0001), and Cluster 3 had significantly lower Factor 1 composite scores compared to Cluster 2 (p<0.0001) (Fig 1).

**Table 4:** Association between patient characteristics and clusters [n(%) or Mean (SD)].

	Cluster 1 (n = 53)	Cluster 2 (n = 59)	Cluster 3 (n = 95)	P-value^Ω^
**Demographics**				
Male	43 (81.1%)	47 (79.7%)	72(75.8%)	0.72
Caucasian	43 (81.1%)	49 (83.1%)	74(77.9%)	0.58
Age of Diagnosis (years)	53.4 (12)	54.7 (10)	53.1(10)	0.65
Ever Smoked	22 (41.5%)	31 (52.5%)	54(56.8%)	0.20
**Diagnosed by Granulomas**	48 (98.0%)	54 (91.5%)	77(81.9%)	0.01
**Lymphocyte Percent**^§^				
Closest to Diagnosis	3.14 (0.13) ^ξ^	3.0 (0.12)	2.72(0.10)	0.03
Most Recent	3.0 (1.0)	2.9 (0.80)	2.8(1.0)	0.39
**Abnormal Chest X-ray**	18 (34.6%)*^ξ^	2 (4.0%)	3(3.0%)	<0.0001
**Treatment Ever**	25(47.2%)*^ξ^	6 (10.2%) ^€^	18(19.3%)	<0.0001
**Exposure**				
Years of Exposure	13.9 (9.7)	16.5 (12.0)	15.3(9.7)	0.42
Machinist vs. Non	9 (17.7%)	6 (12.0%)	25(28.1%)	0.06
High vs. Low	45 (88.2%)	38 (76.0%)	78(87.6%)	0.13
**High-Risk E69**	43 (89.6%)	45 (90.0%)	76(90.1%)	0.99

^Ω^ Comparisons were ANOVA or Pearson’s chi-square tests

^§^Natural logarithm transformed

*Cluster 1 significantly differed from Cluster 2 (p<0.0001)

^ξ^ Cluster 1 significantly differed from Cluster 3(p<0.0001)

^€^ Cluster 2 significantly differed from Cluster 3 (p<0.0001)

### Baseline characteristics of individuals as predictors of cluster membership

Individuals in Cluster 1 and Cluster 2 were more likely to be diagnosed based on having granulomas on biopsy compared to Cluster 3 (Table 4; p = 0.01). Cluster 1 also had a significantly higher proportion of patients who had been treated with steroids and had more abnormal chest x-rays, than Clusters 2 and 3 (p<0.0001, p<0.0001). The third measure, BAL lymphocyte percentage, was higher on average at diagnosis in Cluster 1 compared to Cluster 3, but not cluster 2 (p <0.0001, p = 0.17). Baseline lung function also differed across Clusters based on FEV1, FVC, TLC and DLCO (Table 5; all p< 0.0001); Cluster 1 had the lowest baseline lung function, followed by Cluster 2 and then Cluster 3.

**Table 5:** Association between lung function and clusters.

	Cluster 1 Mean (SD) (n = 53)	Cluster 2 Mean (SD) (n = 59)	Cluster 3 Mean (SD) (n = 95)	P-value
**Factors**				
Factor 1 PFT,+ VO_2_m + WLM	-4.8 (2.3) *^ξ^	4.7(1.6) ^€^	-0.11(1.4)	<0.0001
Factor 2 Exercise Test	-2.7 (3.3) *^ξ^	0.56(1.9)	1.1(1.7)	<0.0001
**Estimate at 30 Years from First Exposure**				
FEV1 (liters)	1.86 (0.40)*^ξ^	3.11 (0.34)^€^	2.6 (.32)	<0.0001
FVC (liters)	2.34 (0.51) *^ξ^	4.03 (0.43) ^€^	3.2 (0.34)	<0.0001
TLC (liters)	4.75 (0.92) *^ξ^	6.46 (0.67) ^€^	5.7 (0.63)	<0.0001
DLCO (mL/min/mmHg)	15.3 (4.2) *^ξ^	29.5 (6.7) ^€^	22.7 (4.7)	<0.0001
(A-a)O_2_ rest (mmHg)	15.4 (5.1) *^ξ^	10.9 (3.15)	10.2 (3.3)	<0.0001
(A-a)O_2_ max (mmHg)	29.6 (16.8) *^ξ^	17.2 (7.7)	14.2 (7.6)	<0.0001
PaO_2_r (mmHg)	66.1 (5.8) *^ξ^	71.6 (4.5)	72.5 (4.33)	<0.0001
PaO_2_m (mmHg)	67.0 (10.5) *^ξ^	76.9 (7.6)	78.5 (6.4)	<0.0001
VO_2_m (liters/minute)	1.11 (0.2) *^ξ^	1.81 (0.27) ^€^	1.38 (0.23)	<0.0001
WLM (watts)	83.6 (20.5) *^ξ^	158.0 (27.1) ^€^	118.5 (22.2)	<0.0001
**Baseline Value**				
FEV1% predicted	76.5 (15.0)*^ξ^	99.7 (14.7)	92.3 (16.1)	<0.0001
FVC % predicted	76.5 (13.8)*^ξ^	97.2 (13.8)^€^	89.0 (13.1)	<0.0001
TLC % predicted	93.7 (14.7)*^ξ^	110.9 (12.3)^€^	103.7 (13.4)	<0.0001
DLCO % predicted	80.2 (24.4)*^ξ^	102.0 (19.2)	92.8 (18.5)	<0.0001

*Cluster 1 significantly differed from Cluster 2 (p<0.0001),

^**ξ**^ Cluster 1 significantly differed from Cluster 3 (p<0.0001),

^**€**^Cluster 2 significantly differed from Cluster 3 (p<0.001)

### Longitudinal models with severity covariates included

Having a job as a beryllium machinist (a surrogate measure for higher exposure),was only marginally significantly different between clusters and was not associated with either factor (Table 6). The results from the individual longitudinal trait analyses were consistent with the cluster and factor analyses that considered all of the lung function variables jointly; steroid use was associated with worse trajectories over time for individual outcomes just as it was associated with clusters and factor values that represent poorer lung function. These results indicate that the composite measures (factors and clusters) give information similar to individual measures while greatly reducing the number of variables that need to be considered in a statistical analysis or disease severity summary for a clinician.

**Table 6:** Longitudinal models of lung function and current severity proxies.

Severity Proxy	Outcome Measure	Time Slope Estimate (SE)	P-value*
Treatment (Yes vs. No)			
	FEV1	0.40 (0.002)	0.0003
	FVC	0.31 (0.15)	0.03
	TLC	0.22 (0.18)	0.23
	DLCO	3.58 (1.4)	0.01
	VO_2_m	0.14 (0.03)	<0.0001
	WLM	19.8 (2.7)	<0.0001
	(A-a)O_2_r	-4.22 (0.51)	<0.0001
	(A-a)O_2_m	-9.4 (0.88)	<0.0001
	PaO_2_m	8.8 (0.86)	<0.0001
	PaO_2_r	4.4 (0.62)	<0.0001
Machinist (Yes vs. No)			
	FEV1	-0.005 (0.12)	0.97
	FVC	0.03 (0.16)	0.87
	TLC	0.007 (0.004)	0.74
	DLCO	0.38 (1.5)	0.79
	VO_2_m	0.06 (0.03)	0.10
	WLM	4.5 (3.0)	0.13
	(A-a)O_2_rest	-1.0 (0.58)	0.07
	(A-a)O_2_max	-0.47 (1.0)	0.64
	PaO_2_m	-0.47 (0.99)	0.63
	PaO_2_r	-0.19 (0.69)	0.78

*All models adjusted for age, sex and race

## Discussion

The purpose of this study was to use data reduction techniques to comprehensively characterize CBD disease phenotypes using longitudinal information. Factor and cluster analyses were performed with longitudinal exercise test and PFT model estimates to reduce the number of outcome measures evaluated individually, and to determine if joint analysis of the results from these longitudinal measures would produce a more comprehensive understanding and assessment of CBD severity. The factor and cluster analyses provide complimentary information to the characterization of CBD phenotype. The factor analysis identified underlying processes being measured by the lung function variables. The cluster analysis grouped the patients in terms of similarity across the measures of lung function in an attempt to find subgroups of patients with similar physiologic characteristics.

We identified two unique underlying factors contributing to the covariance of the multiple measures of lung function among patients with CBD; one factor included all the PFT measures along with two exercise measures, and the other included the rest of the exercise test measures of gas exchange. Patient-specific values for these factors were associated with membership in one of three distinct groups of patients that were identified via clustering of the patients based on similarity across the multiple measures of pulmonary physiology. In addition, both the factor and the cluster membership were associated with traditionally-held markers of CBD severity. Using both of these methods, we found that both the PFT data and exercise test data gave unique and meaningful information towards the characterization of CBD.

We found some key differences in the patient characteristics that predicted the factors and different clusters in our population of CBD subjects. Factor 1 (PFT + VO_2_max and WLM) included the PFT plus two exercise test measures and these measures were the distinguishing characteristics between two of the clusters of patients. These results might suggest that that these physiologic abnormalities help define exercise capacity to a greater extent than the absolute measures of gas exchange during exercise. It is interesting that DLCO, an assessment of gas exchange, was not related with the exercise determination of oxygen levels and Alveolar-arterial gradient, suggesting that it assesses other aspects of lung function. We also found that there were proportionally more patients who worked as machinists and who were on corticosteroids in Cluster 3 compared to Cluster 2, supporting our current concepts of more severe disease among those who are on treatment and those who tend to have higher beryllium exposure. The variable “working as a machinist” is a surrogate of exposure and it is worth noting that when evaluating high and low measures of exposure, both of these clusters had higher exposure, although not statistically significantly different. There were even more patients on treatment in Cluster 1, the cluster with the highest proportion of patients with an abnormal chest x-ray (the most severe group). It is possible that these results could be an indication that treatment is ineffective since Cluster 1 had more severe gas exchange abnormalities.

Our findings also indicate that both the PFT and exercise measures helped to differentiate between the clusters, and that both types of lung physiology tests added unique information to the characterization of CBD. These data support other CBD and sarcoidosis studies suggesting that exercise tests may determine early physiologic abnormalities[24] as well as determine distinct abnormalities in disease.[25] Due to the physical requirements and the need to place an arterial line, some patients are reluctant to complete the exercise testing. However, these results may encourage patients to see the benefits of exercise physiology in disease diagnosis and phenotyping. Future studies, with permutations of these models, may predict patient prognosis based on intermittent exercise testing with more routine lung function data.

In a cross-sectional study of sarcoidosis patients,[26] three measurements from the PFT testing were associated with a sarcoidosis disease severity score developed in that study. More recent studies have evaluated other disease phenotypes with the use of longitudinal measures to better understand disease etiology. In a study of Cystic Fibrosis (CF), longitudinal FEV1 percent predicted estimates at age 20 were the best at differentiating between severe and non-severe groups of patients with Cystic Fibrosis using logistic regression and ROC curves.[27] Other longitudinal Cystic Fibrosis studies have also used FEV1 or FVC to identify severe disease.[28] In our study, we included multiple PFT and exercise test measures, since it is our clinical experience that there are different physiologic abnormalities that result from CBD based on different types of lung impairment. For example, CBD may manifest as an isolated airflow limitation, or a gas exchange abnormality[18, 24] and using multivariate techniques enables better characterization of these and other clinically distinct phenotypes of disease. CBD has historically been thought to be a homogeneous disease, although our studies suggest heterogeneity of disease phenotype. Certainly, CBD is much less heterogeneous than sarcoidosis with only lung involvement evident in contrast to sarcoidosis in which multiple organs can be involved; thus current paradigms for phenotyping sarcoidosis that address other organ involvement besides lung are not applicable to CBD. It appears that the factors and clusters are at least as sensitive at indicating disease severity as examining the longitudinal variables separately. In the future, the possibility of using these developed clinical tools to evaluate associations with disease severity in large datasets with many predictors will decrease the complexity of the analyses and also reduce the number of adjustments for multiple comparisons. This may be especially important as we define genetic and genomic risks for chronic beryllium disease.

## Abbreviations

(A-a)O_2_ max: Alveolar-arterial gradient for oxygen at maximal exercise
(A-a)O_2_ rest: Alveolar-arterial gradient for oxygen at rest
BeLPT: beryllium lymphocyte proliferation test
CBD: chronic beryllium disease
DLCO: diffusion capacity
FEV1: forced expiratory volume in one second
FVC: forced vital capacity
PaO_2_m: partial pressure of oxygen at maximal exercise
PaO_2_r: partial pressure of oxygen at rest
PFT: pulmonary function test
TLC: total lung capacity
VO_2_m: oxygen consumption at maximal exercise
WLM: maximum workload
